# Experimental Study of Flexural Performance of UHPC–NC Laminated Beams Exposed to Fire

**DOI:** 10.3390/ma15072605

**Published:** 2022-04-01

**Authors:** Tieming Zhou, Xingwang Sheng

**Affiliations:** 1 School of Civil Engineering, Central South University, Changsha 410075, China; a15227817155@163.com; 2 National Engineering Research Center of High-Speed Railway Construction Technology, Changsha 410075, China

**Keywords:** ultra-high-performance concrete (UHPC), flexural behavior, fire condition

## Abstract

In recent decades, reinforced-concrete bridges have experienced premature deterioration and other problems during service due to severe environmental effects such as fire and corrosion. Previous studies have shown that the use of ultra-high-performance concrete (UHPC) can improve the durability of bridge structures. In this study, four-point bending tests were conducted on twelve UHPC–NC laminated beams with different UHPC-layer heights and at different temperatures in order to evaluate their flexural performance under fire conditions. The test variables were the UHPC heights (20 mm, 50 mm, 80 mm) and temperatures (20 °C, 200 °C, 400 °C, 600 °C), and the effects on the flexural load capacity of UHPC–NC laminated beams under the influence of these factors were investigated. The test results show that the increase in temperature causes the concrete color to change from grayish blue to white and leads to a significant decrease in the flexural load capacity of the stacked beams. The height of the UHPC layer has an important effect on the stiffness of the stacked beams and delays the formation of local cracks, thus improving the durability of the stacked beams.

## 1. Introduction

With the rapid development of infrastructure worldwide, the number of reinforced-concrete (RC) bridges has increased rapidly. For RC bridges, they are often exposed to many threats including corrosion, fatigue loading, and fire during their service [1,2,3]. Notably, if RC beams are not properly designed, then their material mechanical properties of reinforcement and concrete are severely degraded under the action of fire, and drastic internal-force redistribution occurs in the structure [4,5]. Eventually, the load-bearing capacity of RC beams is significantly weakened and the structural safety is seriously threatened [6]. Therefore, the fire resistance of RC beams has increasingly become a research topic in the field of structural engineering and disaster prevention and mitigation at the domestic and international level.

Currently the most widely used concrete in RC bridges is normal concrete (NC), mainly due to its low cost and convenient construction properties [7,8]. Up to now, much efforts has been devoted to studying the mechanical properties of NC beams after fire exposure. Fire conditions lead to changes in the chemical structure and physical properties of concrete, which significantly affect the mechanical properties of reinforced concrete. Concrete structures are subject to thermal expansion as well as shrinkage, both of which weaken the bond between reinforcement and concrete, resulting in a reduction in the stiffness and load-transfer mechanisms of reinforced-concrete structures [9,10,11,12]. Spalling of concrete is an important phenotypic feature of concrete structures under fire conditions. Spalling is caused by the tensile stresses generated by the high pore pressure that accumulates due to the evaporation of water present inside the concrete under fire conditions. Spalling occurs when such tensile stresses in concrete structures within a cross-section exceed the tensile strength of the concrete itself [13]. Kodur et al. [14] conducted residual-bending-performance tests on reinforced-concrete beams exposed to fire conditions. They concluded that reinforced-concrete beams maintained significant flexural strength even when exposed to high temperatures if the steel temperature was below 500 °C. Khan and Royles [15] concluded that the flexural properties of reinforced-concrete beams decreased significantly as they rose to 200 °C, and decreased to 50% after heating to 775 °C, while the damage mode of the beams changed from shear damage to bending after heating to 600 °C.

UHPC is gradually replacing normal concrete in large-span bridges because of its light weight, high strength, durability, and ability to effectively inhibit the development of deflection and cracks in beams under load. Meanwhile, some scholars have achieved results in the study of the flexural properties of UHPC structures exposed to fire. Banerji et al. [16] investigated the flexural performance of UHPC beams under different load levels and fire-exposure conditions through experiments, and the results showed that high load levels lead to the tensile fracture of beams and help to reduce the pore pressure, and better performance was observed in beams with cooling stages. Li et al. [17] carried out flexural tests of UHPC beams after fire exposure with variables including parameters such as exposure temperature, fiber admixture and aggregate particle size, and the results showed that UHPC beams mixed with steel fibers and polyethylene fibers had better flexural properties. Kahanji et al. [18] carried out fire-exposure tests of UHPC beams under different sustained loads for 1 h and observed that as the load increased, the spalling of UHPC beams was gradually suppressed.

As the application of UHPC in bridges increases, some problems have gradually been identified. Compared with NC, UHPC has the defects of complex preparation, expensive production cost, and large self-shrinkage deformation [19,20,21]. Moreover, it was found that fire is more likely to trigger spalling in concrete with low permeability and relatively high compressive strength [22]. UHPC exhibits excellent compressive strength and durability due to its dense bonding materials (e.g., silica fume and slag), but this dense microstructure also leads to extremely low permeability (two decimal orders lower than conventional NC) [17,23]. Therefore, when UHPC structures are exposed to fire conditions, the vapors inside them do not easily escape and are highly susceptible to fire-induced spalling. It can also be found that RC beams with pure UHPC perform poorly in terms of flexural strength and ductility. Some scholars have proposed the adoption of UHPC and NC in the compressive and tensile zones of the beam, respectively, in order to form UHPC–NC laminated beams. This structure not only reduces the production cost and makes reasonable use of the excellent compressive properties of UHPC, but also allows the allocation of a considerable amount of reinforcement on the tensile side of the beam, which is expected to improve the flexural performance of RC beams [24]. It can be seen that UHPC–NC laminated beams have bright application prospects. However, there is still a lack of exploration of the post-fire-exposure mechanical properties of UHPC–NC laminated beams, and the theory of its post-fire-exposure bearing-capacity calculation has not yet been developed. This means that the safety design of this structure is not well guaranteed, which is not conducive to its widespread application in bridge construction.

To fill the above research gap, the mechanical properties of UHPC–RC laminated beams in fire were experimentally investigated in this study. First, fire tests were conducted on laminated beams with different parameters, and the variables of the beams included the relative positions of UHPC and RC and the exposed temperatures, and the damage modes, load–deflection curves, load-carrying capacity, initial stiffness, and strain distribution of the laminated beams were analyzed. In addition, by considering the strength degradation of UHPC and RC after fire exposure, the quadratic relationship curves of the strength of UHPC and RC at different temperatures were established and compared with the experimental results. This study reveals the cracking and failure modes of laminated beams under fire conditions. The test results provide a reference for the design of bending capacity considering the influence of fire, which makes it more safe, reliable and economical in engineering applications.

## 2. Experimental Program

### 2.1. Material Properties

The lower and upper parts of the laminated beams used in this test were NC and UHPC, respectively. This form of construction can make full use of the excellent compressive properties of UHPC, and at the same time allocate enough tensile reinforcement on the tensile side, so as to effectively improve the bearing capacity of the beams. The mixes of NC and UHPC are shown in Table 1. The constituent materials of the concrete included silicate cement, aggregate, silica fume, high-efficiency water-reducing agent and steel fiber. The grade of silicate cement was 42.5 and the specific surface area was 360 m^2^/kg. The particle size of the aggregate ranged from 0.4 mm to 0.6 mm. The average particle size of silica fume was 0.1 μm and the specific surface area ranged from 15,000 to 20,000 m^2^/kg. The length, diameter and volume of the steel fibers were 13 mm, 0.16 mm and 2%, respectively. The specimens were reinforced with tensile reinforcement of HRB500 grade and stirrup and structural reinforcement of HRB400 grade. The yield strength (*f_y_*), ultimate strength (*f_st_*) and elastic modulus (*E_s_*) of the reinforcements are shown in Table 2.

While each specimen was cast, three NC cube specimens (150 × 150 × 150 mm^3^) and three UHPC cube specimens (100 × 100 × 100 mm^3^) were also prepared in order to test the compressive strength (*f_c_*) and elastic modulus (*E_c_*) of both concretes after exposure to 20 °C, 200 °C, 400 °C and 600 °C. The tests on the high-temperature mechanical properties of the two concretes were carried out according to the specifications [25], and the results are shown in Table 3. Using the compressive strength of concrete at room temperature as a reference value and dividing the reference value by the compressive strength of concrete at other temperatures, the decrease coefficient of the compressive strength of concrete with respect to temperature can be obtained. It can be found that with the rise in temperature, the compressive strength of both types of concrete shows a decreasing trend and the rate of decrease is gradually accelerated.

### 2.2. Specimen Design

**Figure 1:**
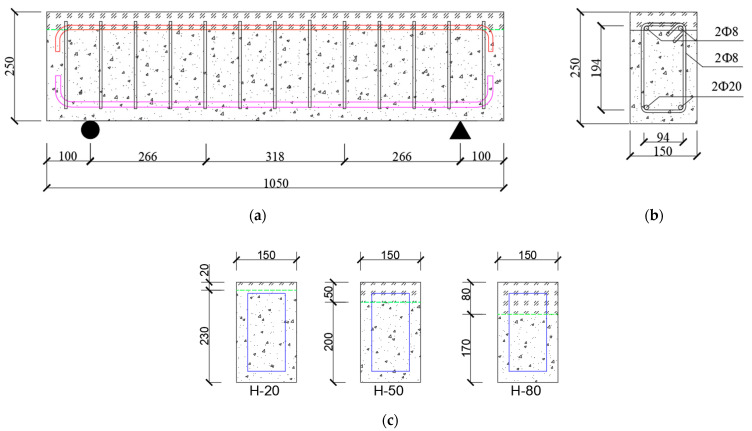
Specimen details: (**a**) front view; (**b**) side view; (**c**) specimens with different UHPC thicknesses (mm).

The specific construction of the specimens is shown in Figure 1a,b. The dimensions of all specimens were 150 mm × 250 mm × 1050 mm. the lower and upper parts of all specimens were constructed with NC and UHPC, respectively. During the specimen casting, NC was cast in the lower part of the specimen first, and then UHPC was cast in the upper part of the specimen. The thickness of the protective reinforcement layer was 20 mm, and the top and bottom of the beam were constructed with structural reinforcement of 8 mm diameter and tensile reinforcement of 20 mm diameter. In addition, 8 mm stirrups were arranged at a spacing of 80 mm. The side view of the specimens with different UHPC thicknesses is shown in Figure 1c. With the UHPC thicknesses (20 mm, 50 mm, 80 mm) and temperatures (ambient, 200 °C, 400 °C, 600 °C) as test variables, a total of 12 specimens were designed and the details of each specimen are shown in Table 4.

### 2.3. Heating and Cooling Treatment

**Figure 2:**
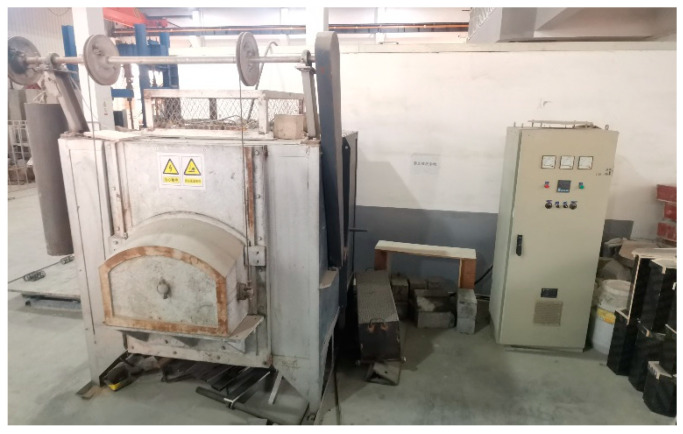
Heating equipment.

**Figure 3:**
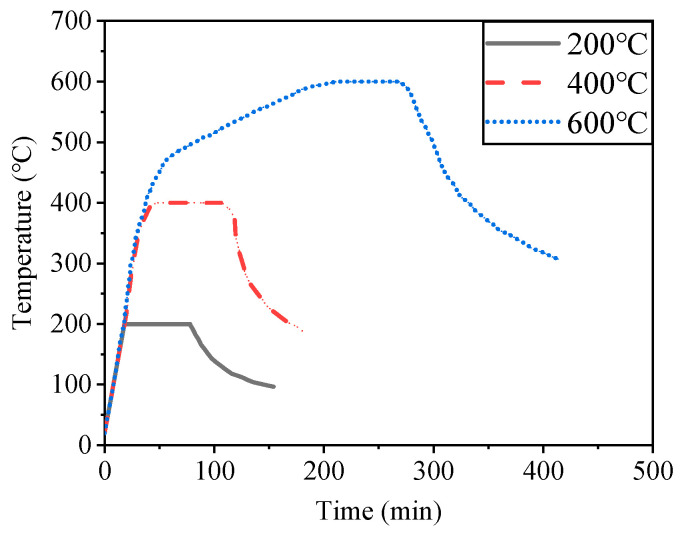
Temperature–time curves.

The heating test of the specimens was carried out in Jiangsu Province, China, and the equipment adopted was an RX3-45-9 resistance furnace as shown in Figure 2. Considering the possibility of the UHPC bursting during heating, the specimens were dried before heating and then put into the heating furnace. In this test, the temperature–time curves shown in Figure 3 were used for the heating and cooling treatment [26]. First, the specimens were heated from room temperature to 200 °C, 400 °C and 600 °C at a rate of 10 °C/min, taking care to monitor the temperature during the heating process. After completing the heating, the specimens were exposed to this temperature for 1 h to make the internal temperature of the specimens consistent with the surface temperature in order to ensure uniform heating, and then the heating was terminated. Finally, the specimens were moved to the ground and naturally cooled to room temperature. Note that during the cooling process, the residual heat of the equipment was prevented from affecting the specimen.

### 2.4. Loading Setup and Measurements System

**Figure 4:**
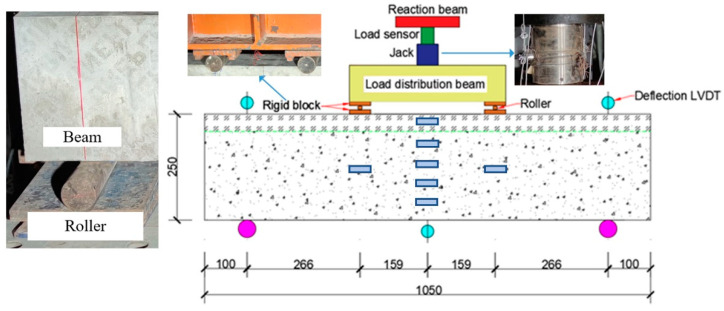
Test setup (mm).

The four-point-bending-test device with the model of YES 500 used in this test is shown in Figure 4. The hydraulic jack is placed between the reaction beam and the distribution beam, and the load applied by the hydraulic jack is measured by a force transducer. The generated load is uniformly transferred to the specimen by means of the steel plates with rollers, and the steel plates serve to prevent stress concentrations from being locally generated in the beam during the loading process. The loading process consists of a preloading phase and a formal loading phase. In the preloading stage, the specimen was preloaded with 80 kN with reference to the calculated flexural load capacity of the specimen, in order to eliminate the gaps and the influence of various unstable factors in the various parts of the loading system. After 10 min of preloading, the readings of each measurement point were recorded as the initial state of the test. In the formal loading stage, before reaching the yield load, force-controlled loading was adopted to load the beam in a graded manner with 40 kN per load level and a holding time of 5 min. After the yield load was reached, displacement-controlled loading was applied at a rate of 3 mm/min per stage. The test was completed when the load was reduced to 80% of the measured maximum load. Throughout the loading process, the concrete cracks appearing on the specimens at each load level were marked with the marker pen.

During the test, concrete strain, reinforcement strain and deflection of the specimens were measured by strain gauges with the model of BF 350 and displacement gauges with the model of KTC 400, and the corresponding data were collected via the TZT3826E strain collector produced by Jiangsu Test Equipment Manufacturing Co., Ltd in Taizhou, China. A total of 7 strain gauges were arranged on the side of the specimen as shown in Figure 4. In the mid-span section, 4 strain gauges were placed at a spacing of 50 mm for the NC layer and 10 mm for the UHPC layer at the top of the specimen. In addition, 2 strain gauges were placed at half the height of the beam at the loading section. Moreover, 3 strain gauges were arranged at a spacing of 65 mm along the transverse direction at the top and 1 strain gauge at the bottom in the span of each specimen. In addition to the strains, the deflections at the span, loading point, and support of the specimen were also measured by displacement gauges.

## 3. Experimental Results and Discussion

### 3.1. Apparent Characteristics after Heating and Cooling

No explosive spalling was observed in any specimen during heating. At room temperature, the color of all test beams was grey blue, and when the temperature was lower than 400 °C, the color of the components exhibited no significant change; when the temperature was higher than 400 °C, the concrete color of the beam began to turn gray and the surface began to crack, but the length and width of the crack are not obvious to the naked eye. At the same time, concrete debris spalling occurs on the beam surface, and the amount of spalling increased with the increase in the heating temperature, but in general the amount of spalling was very small. When the temperature reached 600 °C, the whole beam specimen was white. The reason is that under the high temperature of 600 °C, a large amount of calcium hydroxide decomposes into calcium oxide, which absorbs water and regenerates calcium hydroxide in the process of air cooling. After cooling for a period of time, it precipitates on the surface in the form of crystals. There were many fine cracks on the surface of the beam and the length and width of the cracks increased, accompanied by more concrete debris falling off.

### 3.2. Cracking Patterns and Failure Modes

Due to the limited conditions provided by the furnace, it was not possible to visually monitor the condition of the tested beams during the heating test. However, the observed damage patterns and cracking patterns of the laminated beams after the fire test are valuable for assessing the response of the beams under fire. The results of the load-bearing test for 12 laminated beams after exposure to fire were measured and summarized, and it was found that the majority of the beams were mainly broken by bending, while only the T600-H20 test beam eventually suffered shear failure.

**Figure 5:**
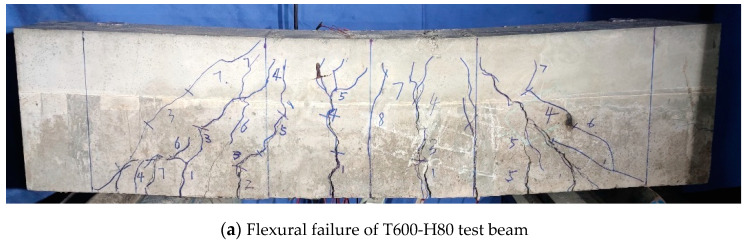

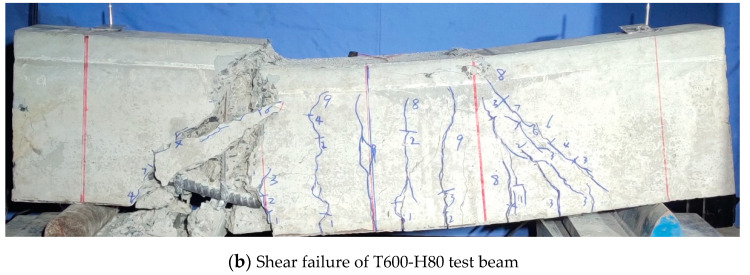
Failure modes of the test beams.

The failure mode and crack development of the test beam with mainly flexural failure are described by taking the T600-H80 test beam as an example (as shown in Figure 5a). The cracks first appeared as vertical cracks in the middle of the span of the laminated beam, when the load was about 60 kN, and as the load increased the number of cracks began to gradually increase and extend upward. However, when the load reached about 80% of the ultimate load-carrying capacity, the cracks extended to the pouring surface of UHPC and the concrete and began to slowly develop. Shortly afterwards, the first cracking sound occurred at the UHPC layer, and then the sound began to occur intensively. Eventually the UHPC in the compressed zone exceeded its ultimate compressive strain, and the UHPC on both sides of the top surface of the span was staggered and caused the beam to be unable to continue to carry the load in the compressed zone, and the beam was finally broken. From the test results, the increase in the height of the UHPC can delay the upward development of cracks. Since the reinforcement in the tensile zone is designed based on overstrength, the UHPC in the compressive zone can bear more pressure on the cross-section due to its good compressive properties, which responds to the material performance advantages of the laminated beam in terms of the tensile resistance of the steel bar and the compressive resistance of the UHPC.

Shear damage only occurred in the T600-H20 of all the test beams (as shown in Figure 5b), and the damage of the specimens was mainly caused by diagonal compression failure due to the development and expansion of the oblique cracks near the bearings. The final damage occurred with diagonal cracks of larger crack width, with an angle of about 45° to the horizontal line. The cracking sequence was as follows: (i) the first vertical crack occurred near the middle of the span and expanded outward from the bottom of the beam; (ii) as the load increased, a set of vertical cracks was generated and expanded toward the shear span of the beam; (iii) when the load reached 40~60% of the peak load, a diagonal crack was formed on top of the vertical cracks in the shear span. Unlike the bending damage of the beam, the damage of the test beam to which shear damage occurred was brittle.

### 3.3. Load-Versus-Deflection Curves at Mid-Span

**Figure 6:**
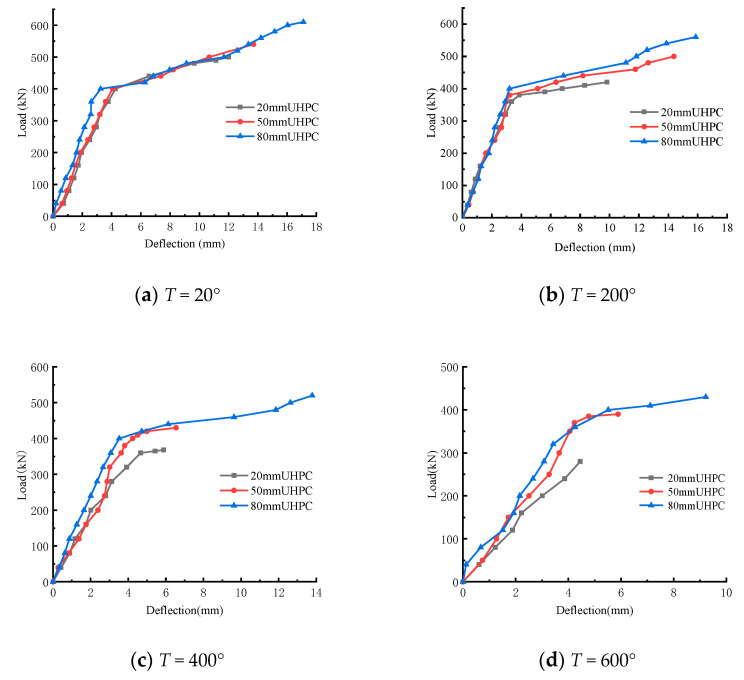
Load–displacement curves at different temperatures.

**Figure 7:**
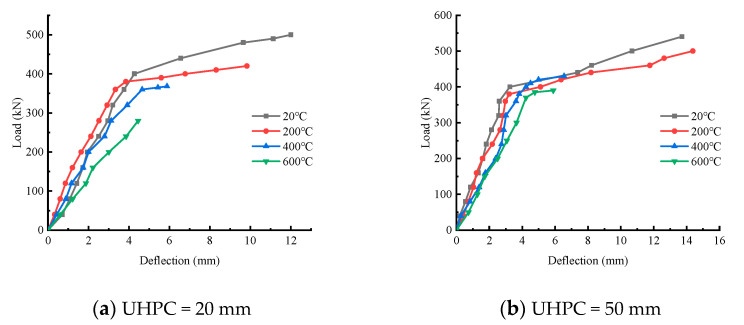

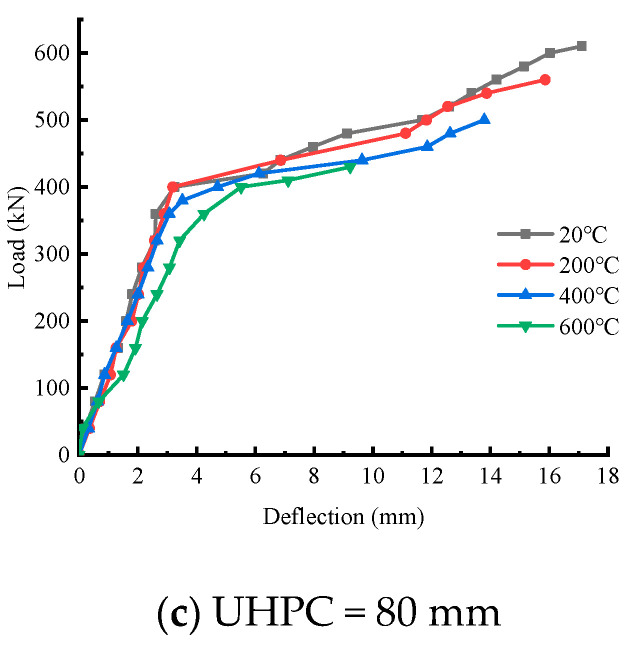
Load–displacement curves at different UHPC heights.

The load–displacement curves of laminated beams at different temperatures and different UHPC heights are respectively shown in Figure 6 and Figure 7, where the displacement is measured in the span of the beam. The load–displacement-variation trend of composite beams under different UHPC thicknesses and temperatures is discussed. It can be seen from the figure that, in general, the load–displacement curve with lower temperature envelopes the load–displacement curve with higher temperature, and the load–displacement curve with larger thickness envelopes the load–displacement curve with smaller thickness of UHPC layer. Except for the shear failure of theT600-H20 test beam, the other eleven test beams were all subjected to bending failure. The load–displacement curve of the former has only one stage, namely the linear elastic stage, during which the load and displacement increase approximately uniformly. With the increase in cracks, before the UHPC concrete at the top of the beam was crushed, the concrete at the bottom of the beam reached the ultimate tensile strain, and the test beam rapidly failed and could not continue to bear the load. The load–displacement curve of the latter can be roughly divided into two stages. The first stage is also the linear elastic stage. In the second stage, with the increase in mid-span deflection, the speed of load growth slows down and gradually reaches the yield load. It can be seen that under the coupling effect of temperature and upper-UHPC-layer thickness, the influence on the failure mode of the test beam is not linear.

### 3.4. Initial Stiffness and Load-Bearing Capacity

**Figure 8:**
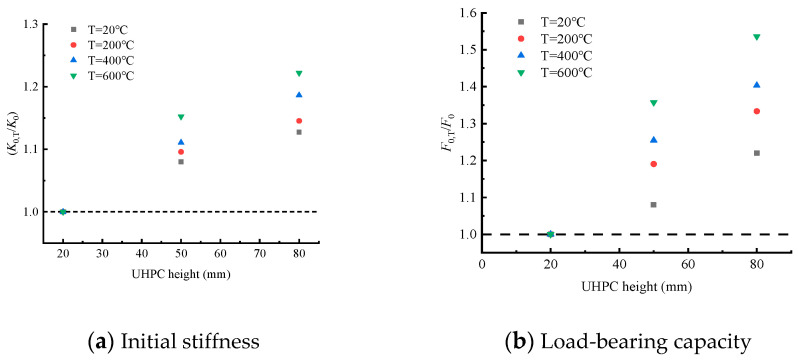
Influence of temperature on load-bearing capacity and initial stiffness of test beams.

Under the action of high temperature, the ultimate load (Pu) and stiffness (K) of the test beam, which control the law of the load–displacement curve, weaken significantly. However, the height of UHPC layer can strengthen the beam, which significantly improves the bearing capacity of the test beam. Figure 8 shows the variation trend of the bearing capacity of the test beam under the action of temperature. Compared with room temperature, the ultimate load (Pu) of the test beam with the same thickness of the UHPC layer was reduced by 20%, 30% and 40% when heated to 200 °C, 400 °C and 600 °C, respectively. This is because the rise in temperature will lead to the loss of the bond and friction of the steel–concrete binder, deterioration of the concrete matrix, the loss of pore water, decomposition of CSH gel and incompatible strain in the steel bar and concrete, thus leading to the loss of the load-transfer mechanism of the test beam. Figure 8 shows the variation trend of bearing capacity of the test beams with different UHPC heights at the same temperature. The ultimate load (Pu) of composite beams at four temperatures increased by 40–60% as the height of the UHPC layer increased. Of all the temperature–UHPC-layer-height combinations considered in the study, it was observed that the combination with the lowest ultimate bearing capacity (T600-H20) had about 46% of the bearing capacity of the combination with the highest yield load (T20-H80).

**Figure 9:**
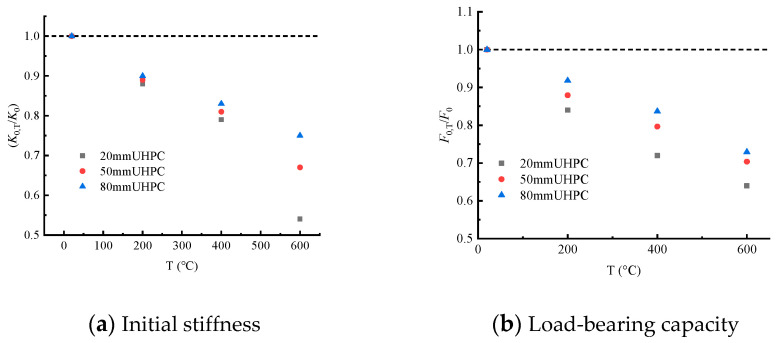
Influence of UHPC height on load-bearing capacity and initial stiffness of test beams.

The secant stiffness corresponding to the yield load of 40% was taken as the initial stiffness of the test beam. Figure 9 shows the influence of the thickness of the UHPC layer on the initial stiffness of each component. At a certain temperature, although the thickness of the UHPC layer increased the stiffness of the beam, the effect was not obvious. Figure 9 shows the influence of temperature on the initial stiffness of the test beam. The initial stiffness of the composite beams conspicuously decreased with the increase in temperature, indicating that the initial stiffness is more sensitive to temperature. The composite beam with the most significant stiffness reduction is T600-H20, which is only 54% of that of T20-H20. Compared with T20-H80, the stiffness of T600-H80 decreased by 25%, indicating that the sensitivity of the initial stiffness of the test beam decreases with the increase in the thickness of the UHPC layer.

### 3.5. Strain Distribution

**Figure 10:**
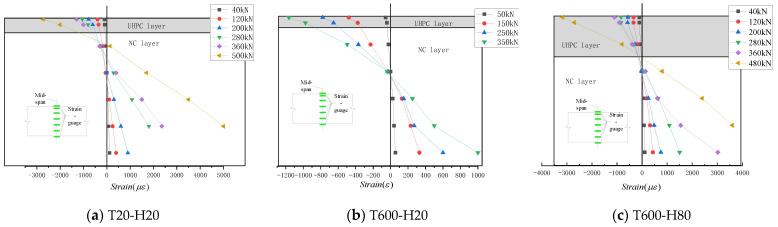
Strain distribution of mid-span.

Figure 10 shows the strain distribution of the concrete surface along the section height in the mid-span of the three test beams (T20-H20, T600-H20, and T600-H80). From the figure, it can be seen that when the load is small at the initial stage of loading, the strain on the concrete surface conforms to a linear distribution along the height and satisfies the assumption of a flat section. From Figure 10a, it can be seen that when the tensile strain of concrete in the tensile zone exceeds the ultimate tensile strain, cracks appear and the tensile strain increases significantly. Near the peak load, the concrete in the tensile zone cracks, leading to the severe failure of the bottom strain gauges. However, due to the good compressive properties of UHPC, in the test beam damaged by bending (as shown in (a) for T20-H20), the top UHPC strain gauges in the span can reach 0.3% at the time of damage. In addition, the test beam underwent a significant upward shift in the neutral axis at the time of failure, reflecting the cracking of the concrete on the tensile side exiting the work. The tensile force of the cross-section was borne by the reinforcement, and the height of the compressive zone was greater than the thickness of the UHPC, which made full use of its excellent compressive properties before the damage. For the T600-H20 test beam where shear damage occurred, the compressive strain of UHPC in the compressive zone at the time of damage was far from the ultimate compressive strain of UHPC (as shown in (b) of Figure 10), which indicates that the compressive zone of the UHPC in the laminated beam did not provide much shear-bearing capacity.

Based on the strain distribution of the cross-section, the height of the compression zone at the time of damage of the 12 beams is listed in Table 5. There is a pattern in the height of the compressive zone with respect to the fire temperature and the UHPC height: as the temperature increases, the height of the compressive zone also increases, and as the height of the UHPC increases, the height of the compressive zone decreases.

## 4. Flexural Load Capacity of UHPC–RC Composite Beam after Fire

There are differences in the fire resistance of UHPC compared to ordinary concrete [27,28], and the flexural bearing capacity of UHPC laminated beams after fire exposure is an important factor affecting the application of UHPC laminated beams in high-temperature fields, but most of the relevant studies on the flexural bearing capacity of UHPC laminated beams at present are focused on the study of room-temperature environments [29,30], and the bearing capacity of UHPC laminated beams after fire exposure is less studied. In this section, the test results of UHPC laminated beams after fire exposure are used to theoretically analyze the UHPC flexural bearing capacity at different temperatures and compare it with the test by introducing the temperature-degradation coefficient of UHPC strength.

**Figure 11:**
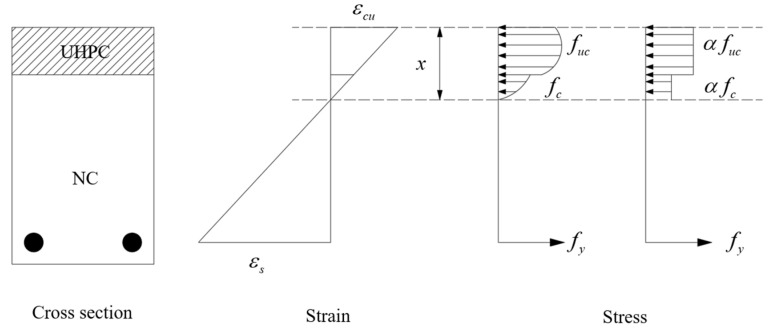
Strain and stress distributions of the beam in the mid-span during failure.

When a flexural failure occurs in the UHPC–NC beam, the strain distribution of the beam cross-section is shown in Figure 11. The stress distribution of the corresponding cross-section can be obtained based on the constitutive model of each material, where $\varepsilon_{s}$ and $\varepsilon_{cu}$ are the tensile strain of steel and compressive strain of UHPC, respectively; *x* is the height of the compressive zone of the cross-section; $f_{y}$ is the stress of tensile steel; $f_{uc}$ and $f_{c}$ are the compressive stress of UHPC and concrete, respectively.

Based on the relationship between the height *x* of the compressed zone and the height *h_u_* of the UHPC, the two cases can be classified as: (a) when *x* > *h_u_*, the pressure in the compressed zone is shared by concrete and UHPC; (b) when *x* < *h_u_*, the UHPC layer is only partially compressed. In fact, the stress distribution of UHPC and concrete is curvilinear. In order to simplify the calculation and to consider the effect of plastic development, the compressive stress of concrete and the compressive-stress distribution of UHPC are both simplified to a rectangular shape. The simplification principle is in accordance with the equal joint forces and equal joint moments, and the simplified stress distribution is shown in Figure 11.

According to existing studies, the residual compressive strength of UHPC and concrete changes after a fire. In order to consider the effect of fire on the flexural bearing capacity of the laminated beam, the strength-degradation factors $k_{u,T}$ and $k_{c,T}$ of UHPC and concrete are introduced to discount the moment bearing capacity provided to the section by UHPC and concrete. Combining the concrete code and Figure 11, the ultimate flexural bearing capacity of the beam is derived from the equilibrium equation of the moment (with the center of gravity of the tensile reinforcement as the axis), and Equations (1) and (2) are obtained.

(1) $$M_{u,T}=\left\{ \begin{array}{ll} k_{u,T}M_{UHPC}+k_{c,T}M_{C} & x>h_{u}\\ k_{u,T}M_{UHPC} & x\leq h_{u} \end{array}\right.$$

(2) $$M_{u,T}=\left\{ \begin{array}{ll} k_{u,T}\alpha_{1}f_{uc}bh_{u}(h_{0}-\frac{h_{u}}{2})+k_{c,T}\alpha_{1}f_{c}b(x-h_{u})(h_{0}-\frac{x+h_{u}}{2}) & x>h_{u}\\ k_{u,T}\alpha_{1}f_{ucu}bx(h_{0}-\frac{x}{2}) & x\leq h_{u} \end{array}\right.$$

where, $M_{u,T}$ is the ultimate flexural bearing capacity of UHPC at fire temperature *T*; $k_{u,T}$ and $k_{c,T}$ are the strength-degradation coefficients of UHPC and concrete at fire temperature *T*; $\alpha_{1}$ is the equivalence factor; $f_{uc}$ and $f_{c}$ are the compressive strengths of UHPC and concrete, respectively; *b* is the section width; *h_u_* is the thickness of the UHPC; *x* is the height of the compressive zone of the section.

Assuming that the correlation with $k_{u,T}$ and $k_{c,T}$ is only temperature dependent and quadratic with the fire temperature *T*, satisfying Equation (3):

(3) $$\begin{array}{l} k_{u,T}=a_{u}T^{2}+b_{u}T+c_{u}\\ k_{c,T}=a_{c}T^{2}+b_{c}T+c_{c} \end{array}$$

The specific steps of the derivation: At first, based on the flexural load capacity of four sets of test beams at different temperatures, the strength-degradation coefficients $k_{c,T}$ and $k_{u,T}$ of concrete and UHPC at different fire temperatures are obtained by back-propagating the load capacity of two beams and verifying their accuracy. Subsequently, a quadratic fitting of *k_T_* according to the fire temperature *T* is performed to yield the parameters to be determined in Equation (3).

The height of the cross-sectional compression zone for each test beam in the damage phase according to the distribution of the test-strain data is given in Table 5. The degradation coefficients were inferred using four groups of beam-bearing-capacity data for different fire temperatures from the fitted data, as shown in Table 5. Among them, beams with UHPC thicknesses of 20 mm and 50 mm were selected as the fitted data for the experimental groups of 20°, 200° and 400°, and 80 mm was used as the error-verification standard, and the error was considered to be less than 5% to satisfy the accuracy. Because the T600-H20 underwent shear damage, the T600-H50 and T600-H80 are the only available data, so the 600° test group has no calculation result.

**Figure 12:**
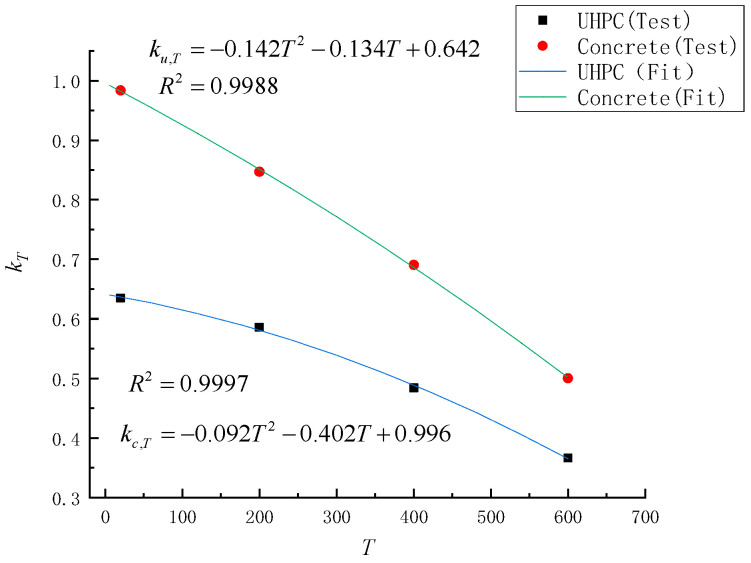
Comparison of fitting results and experimental results.

From the results in Table 6, it can be seen that the strength-degradation coefficients of UHPC and concrete obtained by back-propagation based on 20 mm and 50 mm thicknesses satisfy the error requirements, so the fitting of Equation (2) is performed based on the results in Figure 12. The three parameters to be fitted are *a*, *b* and *c*. The fitted values and standard errors of the parameters to be fitted can be obtained by fitting the strength-degradation coefficients $k_{c,T}$ and $k_{u,T}$ using four sets of data, and the overall effect of the fitting is given by R^2^, and the fitting results are listed in Figure 12.

## 5. Conclusions

This paper investigates the flexural performance of laminated beams under fire conditions. Twelve UHPC–NC laminated beams were tested to investigate the effect of different UHPC-layer heights and different temperatures on the flexural performance of the laminated beams. The following conclusions were obtained from this study.

(1) The results demonstrate that the exposure of the laminated-beam structures to high temperature causes a significant loss of stiffness as well as load-bearing capacity. The increase in the height of the UHPC layer, on the other hand, has an enhancing effect on the laminated beam.
(2) The damage mode of the laminated beam changes under the combined effect of the UHPC height and temperature change, and the effect of the two factors on the damage mode of the laminated beam is not a simple linear relationship.
(3) The test-strain distribution shows that the strain in the top of the beam exceeds the crushing strain limit of the compressive strain of the UHPC, and the crushing of the UHPC can be observed during the damage process.
(4) The quadratic relationship between UHPC and concrete strength and fire temperature was established by introducing the strength-degradation factor and compared with the experimental results with good fitting results.

It is worth pointing out that the flexural performance of laminated beams after fire is explored in this paper. However, shear loads are also common in practical engineering. In addition, there are other extreme factors other than fire, such as corrosion, fatigue loading, etc. Further research in these areas is necessary in the future.

## Figures and Tables

**Table 1:** Concrete mix (kg/m^3^).

Type	Cement	Sand	Silica Fume	Powder		Steel Fiber	Superplasticizer
				Glass	Limestone		
NC	450	992	60	36.3	36.3	110	12.5
UHPC	1026.3	1282.3	128.3	64.1	64.1	156	25.6

**Table 2:** Mechanical properties of reinforcement.

Grade	*f_y_* (MPa)	*f_st_* (MPa)	*E_s_* (MPa)
HRB400	441	636	2.0 × 10^5^
HRB500	522	677	2.1 × 10^5^

**Table 3:** Properties of NC and UHPC.

Type	Temperature (℃)	*f_c_* (MPa)	*E_c_* (GPa)	Decrease Factor
NC	20	51.2	34.6	-
	200	39.8	26.7	0.85
	400	27.3	15.9	0.63
	600	11.1	6.8	0.38
UHPC	20	120.1	24.3	-
	200	100.9	17.8	0.88
	400	85.2	10.5	0.67
	600	61.3	5.4	0.40

**Table 4:** Design parameters of test specimens.

Test Group	Specimen Label	Temperature (°C)	UHPC Thickness (mm)
TB-NT	T20-H20	20	20
	T20-H50		50
	T20-H80		80
TB-200	T200-H20	200	20
	T200-H50		50
	T200-H80		80
TB-400	T400-H20	400	20
	T400-H50		50
	T400-H80		80
TB-600	T600-H20	600	20
	T600-H50		50
	T600-H80		80

**Table 5:** Height of compressed zone of each test beam (mm).

UHPC Height *h_u_* (mm) Fire Temperature *T* (°C)	20	50	80
20	80	73	60
200	85	80	73
400	93	86	76
600	100	90	79

**Table 6:** Degradation coefficients at different fire temperatures.

Fire Temperature *T* (°C)	$k_{u,T}$	$k_{c,T}$	Verification Error (%)
20	0.6352	0.5860	−4.97
200	0.9837	0.8471	−3.60
400	0.6352	0.5860	−3.15
600	0.9837	0.8471	-

## Data Availability

Data are contained within the article.
